# Impact of V-ets Erythroblastosis Virus E26 Oncogene Homolog 1 Gene Polymorphisms Upon Susceptibility to Autoimmune Diseases: A Meta-Analysis

**DOI:** 10.1097/MD.0000000000000923

**Published:** 2015-06-05

**Authors:** Ye Zhou, Miao Liu, Jun Li, Fiza Hashmi, Zhi Mao, Ning Zhang, Liang Zhou, Weiran Lv, Jingwei Zheng, Xiaoli Nie, Changzheng Li

**Affiliations:** From the School of Biotechnology (YZ), Southern Medical University, Guangzhou, China; Department of Physiology and Biophysics (ML, FH), Virginia Commonwealth University School of Medicine, Richmond, VA; Nanfang Hospital (JL, WL, XN, CL), Southern Medical University, Guangzhou; Department of Intensive Care Unit (ZM), Chinese PLA General Hospital, Beijing; First College of Clinical Medicine (NZ), Shandong University of Traditional Chinese Medicine, Jinan, China; Department of Medicine (LZ), Virginia Commonwealth University, Richmond, VA; The Eye Hospital (JZ), Wenzhou Medical University, Wenzhou; and School of Traditional Chinese Medicine (XN, CL), Southern Medical University, Guangzhou, China.

## Abstract

V-ets erythroblastosis virus E26 oncogene homolog 1 (*ETS1*) is recognized as a gene of risk to autoimmune diseases (ADs). Two single nucleotide polymorphisms (SNPs) in *ETS1* (rs1128334 G>A and rs10893872 T>C) were considered associated with ADs risk. However, the results remain conflicting.

We performed a meta-analysis to evaluate more precise estimations of any relationship. We searched PubMed, OvidSP, and Chinese National Knowledge Infrastructure databases (papers published prior to September 12, 2014) and extracted data from eligible studies. Meta-analysis was performed using the STATA 12.0 software. Random effect model or fixed effect model were chosen according to the study heterogeneities.

A total of 11 studies including 7359 cases (9660 controls) for rs1128334 and 8 studies including 5419 cases (7122 controls) for rs10893872 were involved in this meta-analysis. Overall, our results showed that there were significant associations for rs1128334 with AD risk in 5 genetic models, both in pooled analysis and in systemic lupus erythematous (SLE) subgroup, and in 3 genetic models of the uveitis subgroup. Although for rs10893872, the results showed that there were significant associations in allele model both in pooled analysis and in SLE subgroup.

As a conclusion, this meta-analysis demonstrated that these 2 SNPs (rs1128334 and rs10893872) in *ETS1* were associated with ADs risk.

## Abstract 2

Supplemental Digital Content is available in the text

## INTRODUCTION

Autoimmune diseases (ADs) are initiated by abnormal immune response to self-antigen and can result in immune-mediated tissue destruction and chronic disabilities.^1,2^ There are >100 ADs and syndromes, which cause a heavy economic burden in the world, about >$100 billion annually.^3^ More evidence has emerged and showed that genetic background played an important role in the pathogenesis of ADs.^4,5^

The sustained pathology of ADs could be widely regulated by a variety of molecules; V-ets erythroblastosis virus E26 oncogene homolog 1 (*ETS1*) was included as 1 possibility. *ETS1* was the first member of ETS oncogene family, and could regulate tumor development and progression.^6^ Evidence shows that *ETS1* could engage into immunology by downregulating the differentiation of not only B cell but also T helper 17 (T_H_17) cell.^7,8^ Recent articles show that *ETS1* was associated with some types of ADs.^9–11^*ETS1* can be recognized as a risk gene of ADs.

Single nucleotide polymorphisms (SNPs) or mutations in the genetic sequence may alter the expression of the gene.^12–16^ Some researchers paid attention to the relationship between AD risk and 2 polymorphisms of *ETS1*, namely *ETS1* rs1128334 G>A and *ETS1* rs10893872 T>C.^10,17–22^ However, the results remain conflicting. Therefore, we conducted this meta-analysis to make a clarified association between these 2 SNPs and AD risk.

## METHODS

### Publication Search

A systematic search was performed in PubMed, OvidSP, and Chinese National Knowledge Infrastructure databases covering all the papers published before September 12, 2014. The search strategy was as follows: (autoimmune OR autoimmune disease OR autoimmunity) AND (polymorphism OR polymorphisms OR variation OR variations OR mutation OR mutations OR variant OR variants) AND (*ETS1* OR *ETS-1* OR rs1128334 OR rs10893872). The references in these studies were also read to find additional publications on this topic. Articles included met the following criteria: case–control study; evaluation of *ETS1* polymorphisms (rs1128334 or rs10893872) and risk of ADs; available and usable data of genotype frequency.

### Data Extraction

Two authors (Y.Z. and M.L.) independently extracted the data from eligible studies. Data extracted by Y.Z. and M.L. were checked by the third author J.L. The remaining disagreements were discussed and judged by these 3 authors. The following information was extracted: the first author, publication year, diseases, country, genotyping methods, number of cases and controls, the gender distribution of cases and controls, number of genotypes and alleles, Hardy–Weinberg equilibrium (HWE) in control subjects, and the frequency of major allele in controls. Study qualities were judged according to the criteria modified from previous publications^23–26^ (supplementary Table S1, http://links.lww.com/MD/A289).

### Statistical Analysis

Odds ratios (ORs) and 95% confidence intervals (CIs) were calculated as a measure of the association between these 2 SNPs (rs1128334 and rs10893872) and AD risk. Allele model and other different type of genetic models (heterozygote, homozygote, dominant, and recessive) were used. In addition to comparing among all subjects, the stratified comparisons were also used according to different ethnicities and different diseases. The between-study heterogeneity was measured by Cochran (*Q*) and Higgins (*I*^2^) tests. If the heterogeneity was considered significant (*P* < 0.05), the random effects model was used to estimate the pooled OR. Otherwise, the fixed effects model was conducted. Also, logistic meta-regression analysis was carried out, if there was obvious significant heterogeneity, to explore potential sources of heterogeneity. The examined characteristics include publication years, countries, genotyping methods, number of alleles and genotypes, number of female and male patients, and the frequency of major allele in SNP in controls. The HWE was examined using χ^2^ test with significance set at *P* < 0.05. Sensitivity analysis was performed to evaluate the effect of each study on the combined ORs by deleting each study in each turn. Potential publication bias was determined by using Funnel plots and Egger test. An asymmetric plot and the *P* value <0.05 was recognized as significance. All statistical analyses were performed by STATA 12.0 software (STATA Corp, College Station, TX). As a meta-analysis study, ethical approval of this study is not required. This study was reported following the PRISMA guidelines.

## RESULTS

### Study Characteristics

**FIGURE 1:**
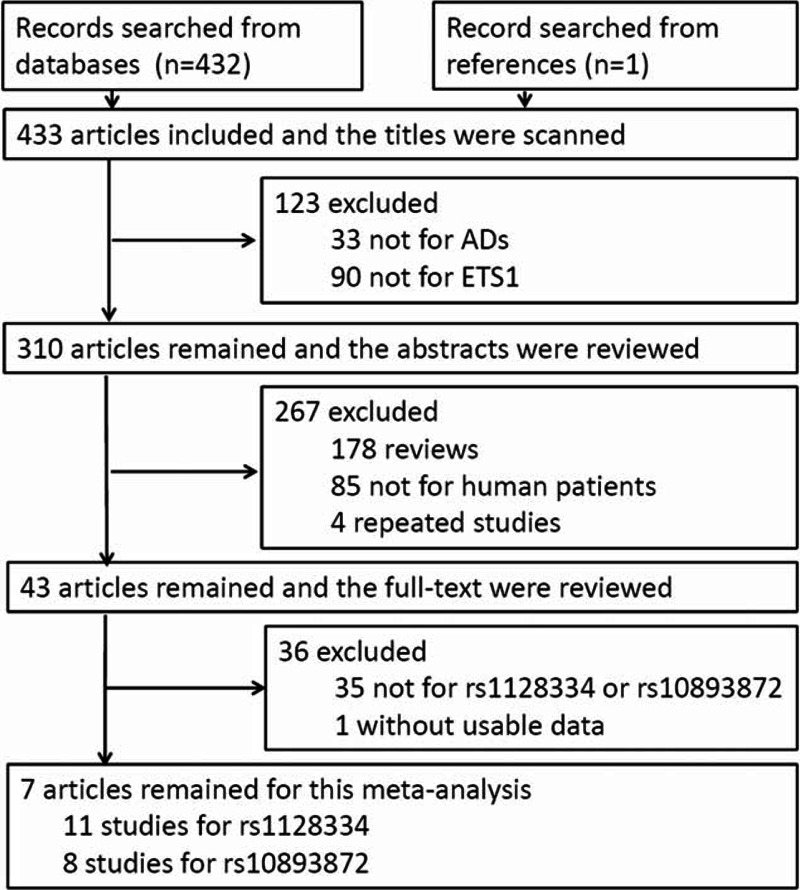
Flowchart for identification of studies included in the meta-analysis. In 433 articles, 33 were found not related to ADs and 90 were found not related to *ETS1* by scanning the titles. After that, 178 articles were recognized as reviews, 85 were found not related to human patients, and 4 articles were repeated papers by reviewing the abstracts. The full-text of the left 43 articles were carefully reviewed, in which 1 article was found not include usable data and 35 articles were found not about rs1128334 or rs10893872. At last, 7 articles remained for this meta-analysis, which included 11 case–control studies for rs1128334 and 8 studies for rs10893872. AD = autoimmune disease; *ETS1* = V-ets erythroblastosis virus E26 oncogene homolog 1.

A total of 432 articles matched the search strategy and an additional article^17^ was found by scanning the references of original papers. After step-by-step screening of the titles, abstracts and full-texts of the articles, as shown in Fig. 1, there were 7 articles appropriate for this meta-analysis, which contained 11 studies for rs1128334, with 7359 cases (9660 controls), and 8 studies for rs10893872, with 5419 cases (7122 controls).

Within all 7 articles, 2 kinds of genotyping methods were used. Only the Asian race was included. The patients in these studies with Behcet Disease (BD), Vogt–Koyanagi–Harada syndrome (VKH), Fuchs uveitis syndrome (FUS), and pediatric uveitis (PU) were all suffering uveitis, which is a common syndrome of ADs. So, these studies were included into uveitis subgroup. There was 1 study not in HWE in control group,^19^ and there was not enough data in another article.^10^ The detail characteristics are shown in Table 1.

**TABLE 1:**
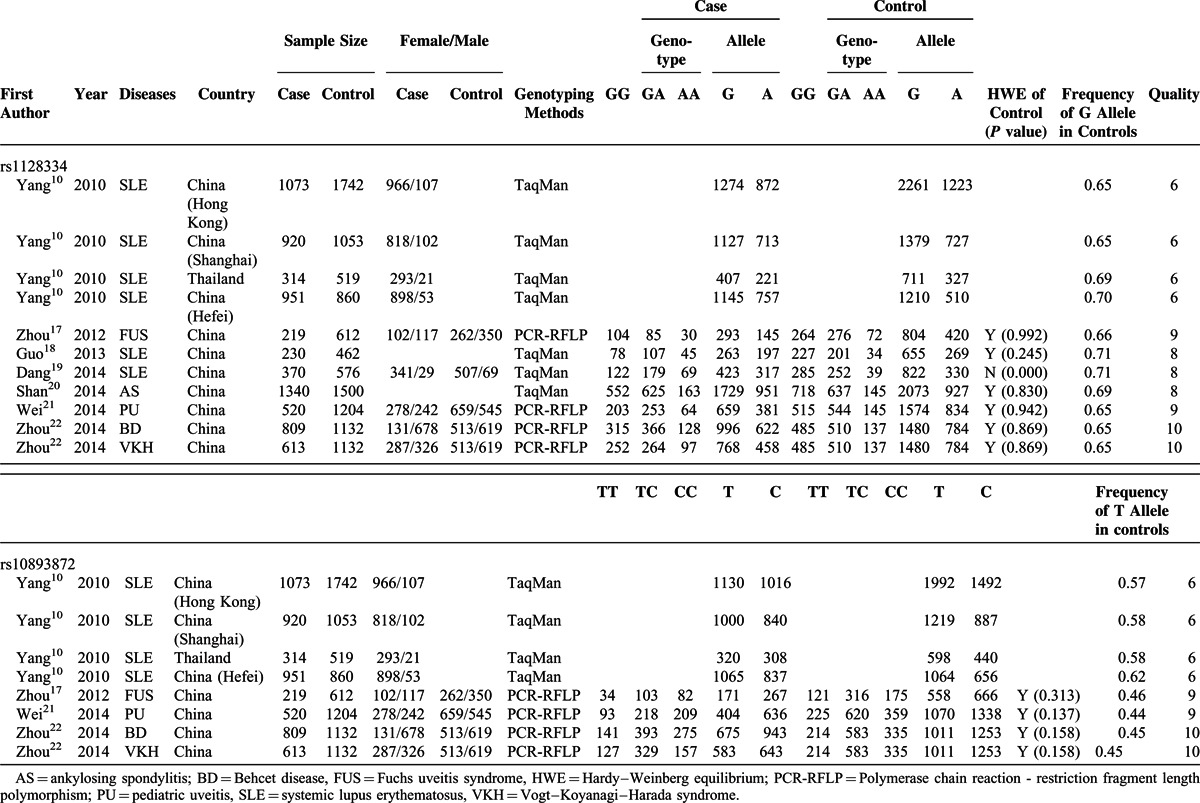
Characteristics of Published Studies of rs1128334 and rs10893872

### Association Between *ETS1* rs1128334 G>A Polymorphism and ADs Risk

**FIGURE 2:**
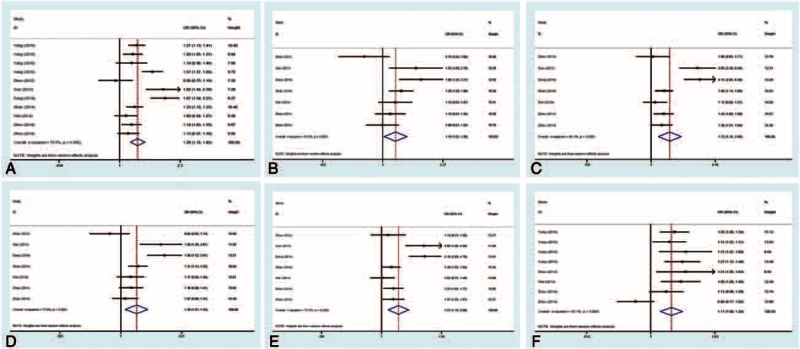
Forest plots of overall analysis of ADs risk associated with *ETS1*. (A–E) Forest plots of overall analysis of ADs risk associated with rs1128334. (A) Allele model, A vs G, random model; (B) heterozygote model, GA vs GG, random model; (C) homozygote model, AA vs GG, random model; (D) dominant model, GA+AA vs GG, random model; (E) recessive model, AA vs GG+GA, random model. (F) Forest plots of overall analysis of ADs risk associated with rs10893872. Allele model, C vs T, random model. AD = autoimmune disease; CI = confidence interval; *ETS1* = V-ets erythroblastosis virus E26 oncogene homolog 1; OR = odds ratio.

First, the association between rs1128334 G>A polymorphism and the risk of AD was analyzed. Significantly increased risks of A allele, GA genotype, AA genotype and GA+AA genotype with ADs were observed in each genetic model in the pooled analyses, respectively (allele model, A vs G, OR 1.28, 95% CI 1.16–1.42, *P* = 0.000; heterozygote model, GA vs GG, OR 1.18, 95% CI 1.02–1.38, *P* = 0.030; homozygote model, AA vs GG, OR 1.72, 95% CI 1.24–2.40, *P* = 0.001; dominant model, GA+AA vs GG, OR 1.28, 95% CI 1.07–1.53, *P* = 0.006; recessive model, OR 1.57, 95% CI 1.19–2.06, *P* = 0.001) (Table 2 and Fig. 2A–E).

**TABLE 2:**
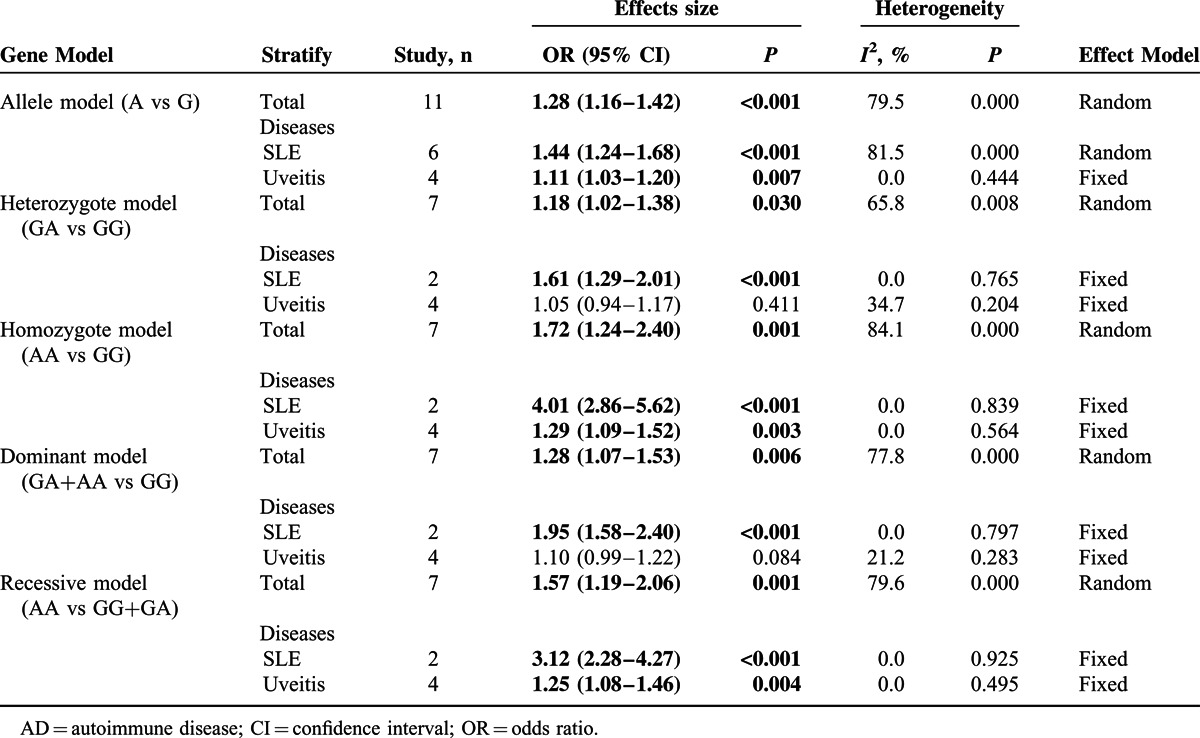
Stratified Analysis of Association Between ADs Risk and rs1128334

Next, we analyzed the studies by subgroup analysis according to diseases. In systemic lupus erythematosus (SLE) subgroup, there were increased disease risks in A allele, GA genotype, AA genotype and GA+AA genotype in each genetic model, respectively (allele model, A vs G, OR 1.44, 95% CI 1.24–1.68, *P* = 0.000; heterozygote model, GA vs GG, OR 1.61, 95% CI 1.29–2.01, *P* = 0.000; homozygote model, AA vs GG, OR 4.01, 95% CI 2.86–5.62, *P* = 0.000; dominant model, GA+AA vs GG, OR 1.95, 95% CI 1.58–2.40, *P* = 0.000; recessive model, OR 3.12, 95% CI 2.28–4.27, *P* = 0.000) (Table 2 and supplementary Figure S1A–E, http://links.lww.com/MD/A289). In the uveitis subgroup, there were increased risks in A allele and AA genotype in allele model (A vs G, OR 1.11, 95% CI 1.03–1.20, *P* = 0.007), homozygote model (AA vs GG, OR 1.29, 95% CI 1.09–1.52, *P* = 0.003), and recessive model (AA vs GG+GA, OR 1.25, 95% CI 1.08–1.46, *P* = 0.004), respectively (Table 2 and supplementary Figure S1F–H, http://links.lww.com/MD/A289).

### Association Between *ETS1* rs10893872 T>C Polymorphism and AD Risk

For the association between rs10893872 T>C polymorphism and AD risk, there was significantly increased risk of C allele in overall comparison in allele model (C vs T, OR 1.17, 95% CI 1.08–1.28, *P* = 0.000) (Table 3 and Fig. 2F). Based on the data limitation, the stratified analysis could only be conducted in the allele model, and the increased risk was found in SLE subgroup (allele model, C vs T, OR 1.22, 95% CI 1.14–1.30, *P* = 0.000) (Table 3 and supplementary Figure S1I, http://links.lww.com/MD/A289).

**TABLE 3:**
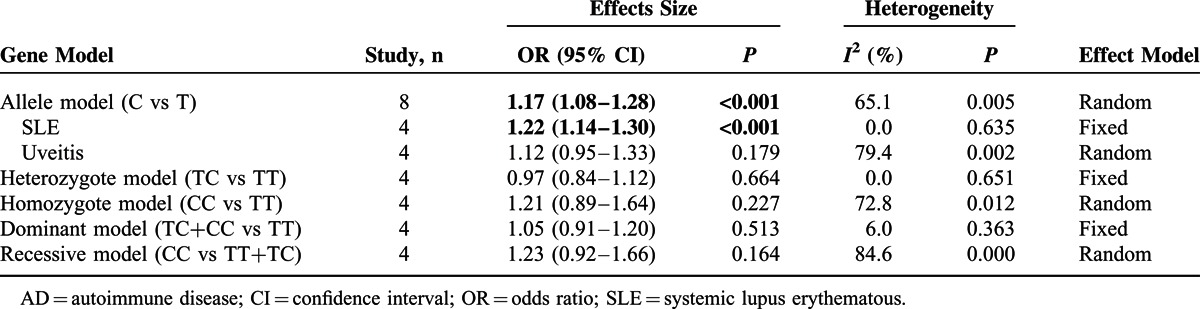
Stratified Analysis of Association Between ADs Risk and rs10893872

### Evaluation of Heterogeneity

The heterogeneities among studies were obvious in the overall comparisons (rs1128334, *I*^2^ = 79.5%, ι^2^ = 0.022, *P* = 0.000; rs10893872, *I*^2^ = 65.1%, ι^2^ = 0.010, *P* = 0.005). The meta- regression analysis was conducted to further explore sources of heterogeneity. Several factors were tested as potential sources of heterogeneity, including publication years, countries, genotyping methods, number of genotypes and alleles, number of female and male patients, and the frequencies of major allele for each SNP in controls. For rs1128334, the genotyping methods (adjusted *R*^2^ = 40.83%) and the frequency of G allele in control (adjusted *R*^2^ = 73.00%) could partially explain the heterogeneity, whereas for rs10893872, the heterogeneity could not be explained by any of the potential sources above.

### Sensitivity and Publication Bias Analysis

**FIGURE 3:**
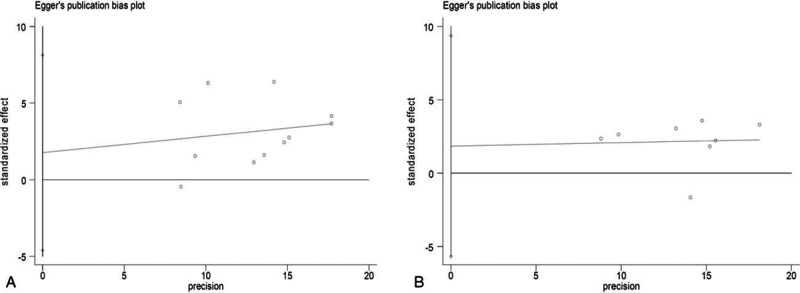
Publication bias on the *ETS1* polymorphism and ADs risk. (A) Publication bias on rs1128334 and ADs risk. (B) Publication bias on rs10893872 and ADs risk. AD = autoimmune disease; *ETS1* = V-ets erythroblastosis virus E26 oncogene homolog 1.

We performed the sensitivity analysis to test the influence of a single study on the overall meta-analysis by deleting each study once a time. As a result, the pooled estimate did not show significant difference (data not shown), which indicated that the results were statistically reliable. No evidence of publication bias was found in current meta-analysis, identified by the Begg test (*P* = 0.640 for rs1128334, *P* = 0.711 for rs10893872) and Egger test (*P* = 0.546 for rs1128334, *P* = 0.569 for rs10893872) (Fig. 3).

## DISCUSSION

*ETS1* is a member of the ETS transcription factor families. It is expressed by a variety of cell types and regulates several functions in some cell signaling pathways.^27^ The differentiation of both B cell and T_H_17 cell could be inhibited by *ETS1*.^7,8^ Animal experiments showed that lupus-like disease could easily be developed in *ETS1*-deficient mice.^28^ Then, *ETS1* was found to be associated with SLE based on human data.^9,10^ As the clinical and immunological overlap of SLE and other ADs,^29^ other researchers found the association of *ETS1* and ankylosing spondylitis (AS).^20^

Some articles reported the relationship between 2 variants (rs1128334 and rs10893872) in *ETS1* and susceptibility to ADs, such as SLE, BD, and VKH.^10,17^ However, the results remain conflicting. Maybe due to different disease types included in ADs, some studies showed that these 2 SNP in *ETS1* were associated with susceptibility to ADs, whereas other studies did not. Therefore, we conducted this meta-analysis, including pooled analysis and subgroup analysis based on different disease types, in order to better understand whether these 2 SNPs contribute to the susceptibility to ADs.

In this meta-analysis, we screened 7 manuscripts and pooled the corresponding data including 7359 cases (9660 controls) for rs1128334 and 5419 cases (7122 controls) for rs10893872. We found that all these 2 SNPs were related to AD risk with distinct degree, respectively.

For rs1128334, A allele, GA genotype, AA genotype, and GA+AA genotype were all found correlated with increased risk of ADs in each genetic model, both in pooled analyses and in SLE subgroup. Moreover, the increased disease risk of A allele and AA genotype were also found in the allele model, homozygote model and recessive model in Uveitis subgroup. For rs10893872, C allele was found to be associated with increased disease risk in allele model, both in pooled analyses and in SLE subgroup. However, there was not any significant association in other genetic models.

There are some limitations in our studies. First, although there were 7 articles included, the studies for some stratified analyses were limited. For example, there were only 2 studies for SLE subgroup in analyses for rs1128334, except in the allele model, whereas there was not enough data to do the stratified analysis for rs10893872 in 4 genetic models, except in the allele model. Also, there was only the data about Asian populations. Further studies based on other ethnic populations will be needed. Second, there were obvious heterogeneities between different groups for some genetic models. Although the meta-regression and sensitivity analyses were conducted, and we found that in rs1128334 the variation of G allele frequency in controls and different genotyping methods could partly explain some heterogeneity, the results still needed to be treated with caution. Third, only 2 SNPs in *ETS1* were included in this study. Some other SNPs in *ETS1* also could contribute to susceptibility to ADs. Not only should the effect of these SNPs, but the interaction or network among these related genes also be studied in the future. Furthermore, studies investigating the gene-environment interactions will also help to make clear of the role of these SNPs in the pathology of ADs. Finally, since ADs consist of diverse diseases, the relationship of these SNPs with other type of ADs, such as rheumatoid arthritis, inflammatory bowel disease and seronegative spondyloarthropathies, should be investigated in the future.

As a conclusion, our study demonstrated that these 2 SNPs (rs1128334 and rs10893872) in *ETS1* confer risk of ADs. Considering the limitation of our study, large sample studies including different ethnic populations and other type of ADs will be needed to confirm the results of this analysis.
